# Assessment of Groundwater Quality in CKDu Affected Areas of Sri Lanka: Implications for Drinking Water Treatment

**DOI:** 10.3390/ijerph16101698

**Published:** 2019-05-14

**Authors:** Titus Cooray, Yuansong Wei, Hui Zhong, Libing Zheng, Sujithra K. Weragoda, Rohan Weerasooriya

**Affiliations:** 1 State Key Joint Laboratory of Environmental Stimulation and Pollution Control, Research Center for Eco-Environmental Sciences, Chinese Academy of Sciences, Beijing 100085, China; titus@uwu.ac.lk (T.C.); zhonghui1977@163.com (H.Z.); lbzheng@rcees.ac.cn (L.Z.); 2 Department of Water Pollution Control Technology, Research Center for Eco-Environmental Sciences, Chinese Academy of Sciences, Beijing 100085, China; 3 University of Chinese Academy of Sciences, Beijing 100049, China; 4 Department of Science and Technology, Uva Wellassa University, Badulla 90000, Sri Lanka; 5 National Water Supply and Drainage Board, Katugastota 20800, Sri Lanka; skwera7@gmail.com; 6 National Institute of Fundamental Studies, Hanthana Road, Kandy 20000, Sri Lanka; rohan.we@nifs.ac.lk

**Keywords:** drinking water, hardness, fluoride, DOM, CKDu

## Abstract

This study investigated the water quality of the groundwater that was collected from the chronic kidney disease of unknown etiology (CKDu) prevailing areas in the dry zone of Sri Lanka to assess its suitability for drinking purposes, and for the first time a Water Quality Index (WQI) with emphasis on proposing appropriate drinking water treatment method was developed. A total of 88 groundwater samples were collected in dry (December 2016) and wet (May 2017) seasons; high concentrations of water hardness, fluoride, salinity, dissolved organic carbon (DOC), and the general alkaline nature of water were the main issues that were observed for disease incidence. The chemical weathering of the underlying bedrock, followed by ion exchange and precipitation processes, primarily controlled groundwater geochemistry. During the 1985–2017 period, the variations of the annual rainfall and temperature were minimal, which suggests no evidence for major climatic changes within the study areas. Almost all of the samples from the CKDu regions show a low alkali hazard and most of the samples show a medium to high salinity hazard. The DOC of the studied samples was mainly composed of the organic fractions in the following order, as fulvic acids > humic acids > aromatic protein II > soluble microbial by-products, and the molecular weights (MW) of these fractions ranged from 100–3000 Da. Based on the water quality index (WQI) calculations, it was found that only 3.8% in the wet season and 2.6% in the dry season of total water samples were categorized as the “excellent” type, and all other water sources require a further treatment before consumption. As there is an urgent need for establishing proper long-term drinking water treatment technology for the CKDu affected area, these findings can be used as benchmark of raw water quality in the design processes of treatment plants.

## 1. Introduction

An increasingly serious global health concern is emerging as the chronic kidney disease of unknown etiology (CKDu), which presents as a kidney disease in patients who do not exhibit common causative factors, such as diabetes or hypertension [1]. The CKDu is prevalent among agricultural workers, and its symptoms include fatigue, panting, lack of appetite, nausea, and anemia [2]. Geographic “hot spots” of CKDu have emerged in a number of countries, including El Salvador, Guatemala, Mexico, Nicaragua, Bulgaria, Croatia, Serbia, India, and Sri Lanka [3]. An increasing number of Sri Lankans suffer from CKDu since the 1990s, e.g., more than 50,000 patients diagnosed with late-stage kidney disease [3,4], and majority of these patients are reported from the North Central (NCP) and North Western (NWP) Provinces of Sri Lanka (Figure S1, Supplementary Material) and a notable number of patients are also reported from the Uva, Eastern, and Northern Provinces [4,5]. Sri Lankan CKDu patients include both men and women in between 17–70 years of age, and the majority of them belong to men aged 30 to 60 [4,5,6], who are in their most productive working age. Therefore, CKDu has become a major environmental health issue that affects both social and economic aspects of the people, even with the availability of free public health services to the entire nation.

Although the CKDu becomes prominent and its causes are not yet known, most of the external factors that trigger the disease are likely associated with the drinking water. Therefore, the importance of “water factor” for disease etiology is hypothesized [4]. Various authors have correlated water quality and peculiar CKDu prevalence without inclusive discussions. The most commonly proposed CKDu causative pollutants are the hardness and fluoride in water [7,8,9]. In most cases, the presence of excess fluoride and hardness (commonly [Ca^2+^] > [Mg^2+^] with major ions ([HCO_3_^−^] > [Cl^−^] > [SO_4_^2−^]) in water seems to aggravate the prevalence of CKDu [10]. In a parallel study, our researchers have fractionated the dissolved organic carbon (DOC) in groundwater from CKDu endemic areas into three groups, as Fraction-I (MW < 900 Da), Fraction-II (900–1800 Da), and Fraction-III (1800–4000 Da); fraction-II is denoted as the building blocks of non-biodegradable organic substances that are mostly enriched in the groundwater of high CKDu prevalence zone [11]. An urgent strategic planning is required to provide water for drinking and cooking facilities for CKDu affected families. Additionally, the provision of household filtration units or other facilities requires close scrutiny [12].

It is well known that water quality is the key to select appropriate water treatment technology, and the water quality index (WQI) is an important tool in determining the drinking water quality in urban, rural and industrial area. Hither to date, the assessment of water quality for drinking or other purposes is made on the basis of discrete set of parameters, as specified by regulatory agencies. However, the availability of a composite index based on individual parameters is convenient for rapid demarcation of water usage. Presently we are interested to develop a composite index for potable water in Sri Lanka. The water classifications that are based on Piper or Stiff diagrams yield different sub water groups. In both methods, only major ions are considered and the classified water type does not provide information on the status of water quality. The availability of a composite index (WQI) is convenient in rapid screening to recommend appropriate water treatment technology for providing safe drinking water. However, none of those previous studies has evaluated the overall water quality of groundwater using the WQI method in the CKDu prevailing area of Sri Lanka to assess the suitability of groundwater for drinking purposes.

The availability of surface water sources are limited, as most of the CKDu affected areas are located in the dry zone (Figure S1a) of the country; therefore, people rely heavily on groundwater sources for their daily needs. Groundwater occurred mainly in two aquifer forms; shallow regoliths and fissures and fractures in hard rocks, and they are extensively used for groundwater extraction [13]. Owing to the underlying high density metamorphic rocks, these aquifers remain as pockets that are isolated with each other and they are recharged by rain water through fissures and crack of the rocks. The dry zone tank cascades (reservoirs), with around 18,000 tanks, have significant control of the recharge of these shallow regolith aquifers. These tanks are clustered in to 3500–4000 cascade systems and they are concentrated in the north central (mainly Anuradhapura district) and north-west zone districts of the country [14,15]. For the drinking water requirements, communities rely on dug wells (maximum depth 15 m build on regolith aquifers) and tube wells (maximum depth 60 m build on hard rock aquifers) of variable depth, depending on the region and the underlying bedrock condition. However, the provision of safe drinking water supply for people is of utmost important, as recommended by the World Health Organization (WHO) for CKDu preventive measures [4]. As a result, small scale low pressure reverse osmosis (RO) plants were established in these areas since 2011. Presently, over 500 small scale (0.5 m^3^/day–3 m^3^/day of treated water) RO drinking water treatment stations are in operation in the CKDu prevailing areas, which sell water at the nominal price (US $ 0.01/L) or provided for free (with the support from individual donors or organizations). As most of the plants are operated by different individuals and/or community organizations, no two plant necessarily follow the same protocols in plant operation, maintenance, and management [16]. During rainy seasons, people tend to use rain water for consumption, which results in a considerable reduction in water demand/production occurring in most of these RO plants during the wet season. In addition, DOC is a typical membrane foulant that contributes to reversible and irreversible membrane fouling [17], thus its mitigation should seriously be considered in the usage of RO drinking water plants. 

Therefore, the objectives of this study were to investigate groundwater quality in the CKDu affected area analyzing inorganic species and DOC for the samples that were collected during different climatic seasons. We calculated the composite water quality index (WQI) based on WHO guidelines and Sri Lankan Water Quality (SLS614:2013) Standards [18], for rapid screening and (if required) recommending appropriate water treatment technologies to meet the requirements for drinking water standards. The WQI calculations were carried out utilizing major and minor species and DOC in water, which imparts serious implications on water palatability.

## 2. Materials and Methods

### 2.1. Sample Collection

The water samples were collected from different CKDu prevailing Divisional Secretariat Divisions of Anuradhapura district (Figure S1b) (high = 35, moderate = 26, and mild = 17), which have the highest number of CKDu patients according to the CKDu data of Ministry of Health, Sri Lanka. Padaviya (7), Kebithigollawa (8), Medawachchiya (10), and Kahatagasdigiliya (10) were selected for high CKDu prevalence divisional secretariats, Mihintale (8), Talawa (7), and Nochchiyagama (11) were selected for moderate CKDu prevalence divisional secretariats, while Galnewa (9) and Palagale (8) were selected for mild CKDu prevalence divisional secretariats. For comparison, five samples were collected from Monaragala (none endemic) and Kandy (CKDu none prevalence) district, respectively. An effort was made to obtain a uniform distribution of sampling locations through the study area as much as possible, but, due to the uneven distribution of population and CKDu patients, this was difficult. Two sets of samples were collected from each location in 2016 December (wet season in Anuradhapura) and 2017 May (dry season in Anuradhapura). All of the sample points were shallow (5–10 m deep dug wells) or medium deep (20–30 m deep hand pump tube wells) wells used for groundwater extractions, and three samples were collected from natural springs. 

### 2.2. Analytical Procedures

All of the samples were collected in pre-washed Teflon bottles filtering through 0.45 µm disposable polyethersulfone membrane syringe filters (Jinteng, Tianjin, China). The samples collected for cation (such as Na, K, Al, Ca, Mg, Fe) analysis were acidified with ultra-pure (GR) nitric acid (Sinopharm, Shanghai, China). All of the samples were stored at 4 ℃ until shipping to China for chemical analysis. In situ determination of pH, electrical conductivity (EC), water temperature was measured with filed probes (WTW, Welheim, Germany), hand held turbidity meter measured turbidity (Hach, Loveland, CO, USA), and alkalinity was measured with Hach© Alakalinity test kit (TNTplus Vials, Loveland, CO, USA). Na, K, Ca, and Mg concentrations were measured by an inductively coupled plasma optical emission spectrophotometer (Optima 8300, Perking Elmer, Houston, TX, USA) instrument, while other tracer cations were analyzed by an inductively coupled plasma mass spectrophotometer (NexION 300X Perking Elmer, Houston, TX, USA) instrument, and all of the anions were analyzed by an ion chromatography (ICS-1000, Dionex, Sunnyvale, CA, USA) instrument. Hardness concentration was calculated based on the Ca and Mg results for each sample. Dissolved organic carbon (DOC) concentrations of the water samples were analyzed by a Vario TOC Select (Elementra, Langenselbold, Germany) instrument. The fluorescence properties of DOM using 3D Excitation Emission Matrices (3D-EEM) was investigated by a fluorescence spectrophotometer (F-7000, Hitachi, Tokyo, Japan). The spectral data were first normalized against water Raman scatter, which was determined daily. Subsequently, water blank matrices were subtracted from the sample matrices to eliminate Raman scatter. Fluorescence intensity was converted to equivalents of quinine sulfate dehydrate by a calibration factor determined from the fluorescence intensity at $\frac{\lambda_{ex}}{\lambda_{em}}=\frac{350}{450}$ [19]. The molecular weight (MW) distribution of DOC was measured by a high-performance size-exclusion chromatography (HPSEC, Breeze 1525, Waters, Milford, MA, USA) instrument that was equipped with a silica gel column (TOSOH, TSK-gel G3000SWXL) using 0.015 M phosphate buffer solution (0.00255 M NaH_2_PO_4_, 0.00245 M Na_2_HPO_4_, and 0.01 M NaCl) at pH 6.6 as mobile phase.

### 2.3. Water Quality Index (WQI) Calculations

The Water Quality Index (WQI) [20] is used in this study to account for the synergy of individual water quality parameters. WQI is single numerical figure that is defined as a rating reflecting the composite influence of different water quality parameters. The WQI values can be mainly calculated by two methods: in the first way, 0–100 scaling was used to classify the water sub-groups [21]. In the other method, the WQI calculations were made without imposing an upper limit [22]. In both of the methods, sub-groups division was operationally done. Presently, we used WQI method without an upper limit to classify our water samples. To compute the WQI, weights (*w_i_*) were assigned to pH, TDS, total hardness, total alkalinity, Ca^2+^, Mg^2+^, Na^+^, F^−^, Cl^−^, SO_4_^2−^, NO_3_^−^, and Fe while considering Sri Lankan standard specified for potable water (SLS614:2013) [18] and WHO guidelines for drinking water quality [23] (Table S1). DOC was included in the WQI calculation when considering the formation of disinfection byproducts [24]. The maximum weight 5 was assigned to TDS, fluoride, nitrate and DOC considering their ability to causing diseases, and then other parameters were given respective weights that considered their relative significance. Subsequently, the relative weights (*W_i_*) of the chemical parameters were computed using the flowing equation.

(1) $$W_{i}=\frac{w_{i}}{\sum_{i=1}^{n}w_{i}}$$

where, *W_i_* is the relative weight, *w_i_* is the weight of each parameter, and *n* is number of parameters. Table S1 provides the calculated relative weight (*W_i_*) values of each water quality parameter. After that, a quality rating scale (*q_i_*) for each parameter was calculated using the following equation.

(2) $$q_{i}=\frac{C_{i}}{S_{i}}\times 100$$

where, *q_i_* is the quality rating, *C_i_* is the concentration of the each chemical parameter in water sample in mg/L, and *S_i_* is the Sri Lankan drinking water standard for each chemical parameter in mg/L. Finally, WQI was computed using *q_i_* and *W_i_*, as shown in below equation.

(3) $$WQI=\overset{n}{\underset{i=1}{\sum }}W_{i}q_{i}$$

Later, the water samples were categorized into different water types (based on the computed WQI value), as mentioned in Table S2. 

## 3. Results and Discussions

### 3.1. Chemical Characteristics of Groundwater

The geochemistry of groundwater is controlled by factors, like rock type, residence time, previous composition of the groundwater, and the presence of other attributes along the flow path [25]. Table 1 shows the average geochemical composition of groundwater in two macro climatic seasons, Table S3 illustrates the composition of sample ensemble of groundwater. All of the samples were slightly alkaline, while some were slightly acidic (pH 5.7) and few were more basic (pH 8.8). The electrical conductivity (EC) values varied from 35 μS/cm to 2890 μS/cm in all areas; throughout the Nochchiagama area, the samples show the highest electrical conductivity values (average 1436, n = 11), due to their proximal locations to arid climatic zone (Figure S1). The ions that leached into groundwater from rock-water interactions may be further concentrated due to high evaporation prevails in dry climatic zone providing high electrical conductivity values. In par with previous data [26], water quality of natural springs is better when compared to other groundwater sources (Table S3 and Table S4). The incident of CKDu among the people who consume spring water is low [26]. However, there is no apparent difference of the quality of water received from dug wells or the tube wells (Table S4).

Cl^−^ is the dominant anion and the anionic contents vary as Cl^−^ > SO_4_^2−^ > F^−^ > NO_3_^−^ > PO_4_^3−^ in all of the groundwater samples investigated. On the other hand, Na^+^ is the most predominant cation and the cations vary as Na^+^ > Ca^2+^ > Mg^2+^ > K^+^. The highest concentrations of Cl^−^ and Na^+^ were observed in the Nochchiyagama area, due to high evaporation rates. When compared to water in the non-prevalence zone, a higher concentration of electrical conductivity, total hardness, and fluoride are recorded in all other CKDu prevailing zones (mild, moderate, and high). Fluoride is a primary (health related) contaminant that requires stringent regulatory protocols to be met prior to water consumption. However, electrical conductivity and hardness are secondary (non-health related) standards; hence, the achievement of regulatory limits of these parameters is not mandatory. However, the excess electrical conductivity and hardness impart serious water palatability issues, which result in their indirect impact on health due to limited water consumption by people. Therefore, any drinking water treatment method should first improve the palatability by regulating hardness and electrical conductivity. Once these conditions are met, excess fluoride (if any) or any other trace constituents require a treatment. Temperature variations over 30 years (1985–2017) are minimal and any major impacts on region water cycle due to climatic changes are unlikely (Figure S1-C1).

As shown in Table 1, electrical conductivity, alkalinity, hardness, fluoride, and many other ionic components are present in higher concentrations in water collected during dry climatic season when compared to those values in the wet climatic season. As shown previously [11], the water DOC levels are high in water of tropical terrains, which is largely due to enhanced microbial activities. As illustrated in Table 1, the DOC levels in water collected during the wet climatic season are higher than the water in dry climatic season. The rainwater acidity seems to play a pivotal role in dissolving terrestrial organic matter in aquifer. Notably, in all zones (except spring waters), the dissolved iron concentration in water collected during wet climatic season is almost tenfold higher than the water in the dry climatic season. It is inferred that the high DOC may enhance mineral Fe (III) reduction into Fe (II), as [27].

Fe^3+^ + Organic matter →Fe^2+^ + CO_2_ + H_2_O + new Organic matter

**Figure 1:**
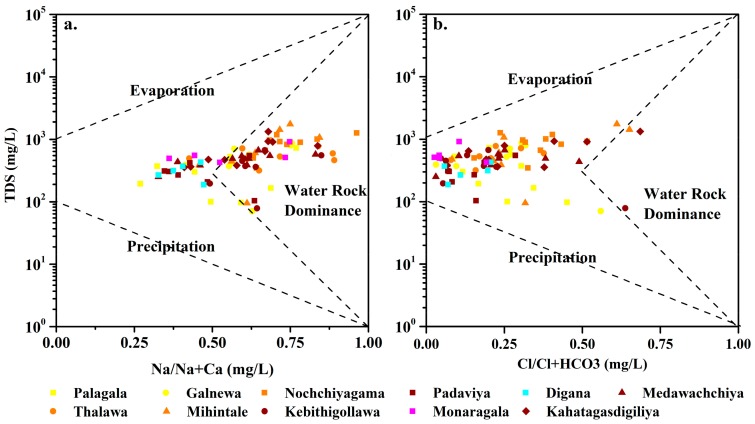
Gibbs plots of groundwater samples from different chronic kidney disease of unknown etiology (CKDu) prevalence areas in wet season. (**a**) Variation of weight ratio of Na/(Na+Ca) as a function of total dissolved solids, (**b**) variation of weight ratio of Cl/(Cl+HCO_3_) as a function of total dissolved solids

The Gibbs’s plot [28] is widely used to describe the sources of dissolved chemical constituents in water contributing from geology, precipitation, and evaporation. As shown in Figure 1, evaporation processes influence the water composition of all zones, but the water–rock interactions mostly control it (Figure 1 and Figure S2). The results further imply that the weathering of underlain metamorphic rocks and subsequent ion exchange reactions mainly control groundwater geochemistry. Therefore, in agreement with previous data [29], the ion exchange and reverse ion exchange are the main possible processes that contribute major ionic constituents to groundwater. The variation of fluoride levels is attributed to the weathering of fluoride-rich mineral phases, like amphiboles, biotite, and apatite, which are ubiquitous in the high grade metamorphic terrains. 

**Figure 2:**
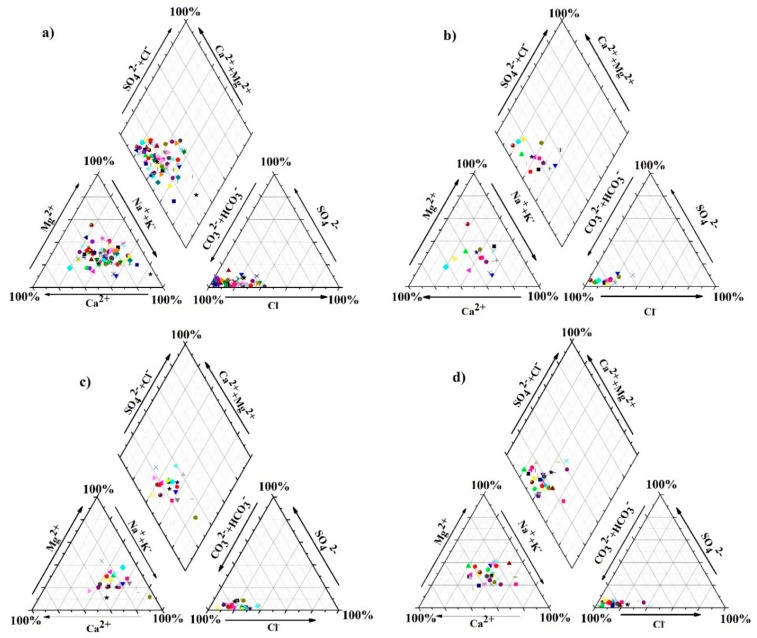
Piper trilinear diagrams showing the different chemical types of groundwater in wet season: (**a**) all samples, (**b**) samples of mild chronic kidney disease of unknown etiology (CKDu) prevalence areas, (**c**) samples of moderate CKDu prevalence areas, and (**d**) samples of high CKDu prevalence areas.

Hydro-geochemical facies in CKDu prevalence zones are demarcated using Piper trilinear diagrams [30] and the following water types were identified (Figure 2): Ca-HCO_3_ type, Mg-HCO_3_ type, and non-dominant cation (NDC)-HCO_3_ type. All of the water types are characterized by the presence of the HCO_3_^−^ group. However, the variations of water types (if any) among CKDu prevailing zones and macro climatic seasons are often obscured. The incongruent dissolution of minerals, like amphiboles and pyroxenes in the aquifer, followed by ion exchange processes contributes high Ca^2+^ and Mg^2+^ concentrations in groundwater [10,31].

**Figure 3:**
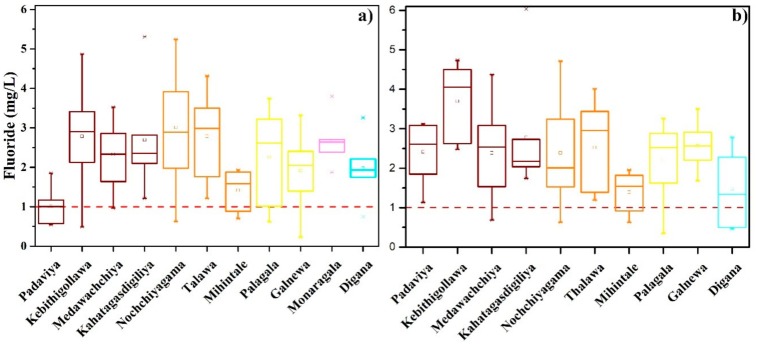
Box and whisker plot showing fluoride distribution in the samples of the wet season (**a**) and dry season (**b**) in study areas. The maximum allowable fluoride concentration at 1.0 mg/L of the SLS 614-2013 for potable water is shown. Different colours represent sampling areas, “*” denotes the maximum/minimum observations.

**Figure 4:**
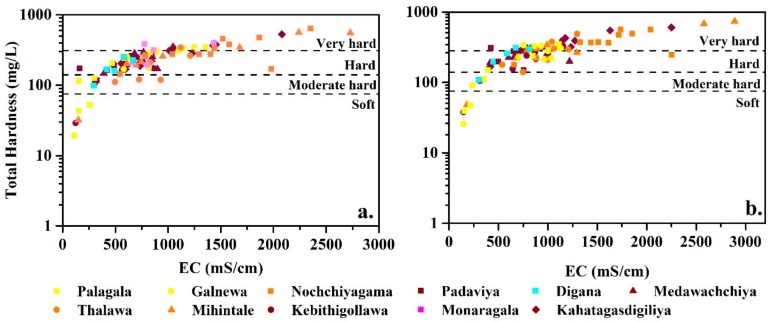
Relationship of hardness and electrical conductivity (EC) of groundwater from study areas in the (**a**) wet season and (**b**) dry season.

In all zones, the fluoride concentrations ranges from 0.2 mg/L to 6.0 mg/L; when compared to the Sri Lankan portable water guidelines, the fluoride levels in groundwater are higher (Figure 3). The highest concentration of fluoride (6.01 mg/L) was recorded in the Kahatagasdigiliya (Figure S3a) area, while the lowest in Galanewa area (0.2 mg/L). In all zones, the climatic variations on fluoride values are not significant (at p < 0.005). Na^+^ controls the availability of fluoride in the groundwater systems showing a strong correlation with fluoride [10]. Even discrete fluoride concentrations did not show any apparent relationship with the CKDu prevalence [7,32]. The synergy between high fluoride and hardness contents is a significant hydro-geochemical feature in high CKDu prevailing zones (hardens 29–608 mg/L, CaCO_3_ and fluoride 0.5–6.0 mg/L), while the levels of fluoride and hardness are comparably low (hardness 19–350, CaCO_3_ mg/L and fluoride 0.2–3.7 mg/L) mild CKDu prevailing zones. However, hardness values were more or less in similar ranges in both wet and dry seasons (Figure 4), while at some instances both hardness and electrical conductivity (EC) values were slightly higher in dry season compared to the wet season.

**Figure 5:**
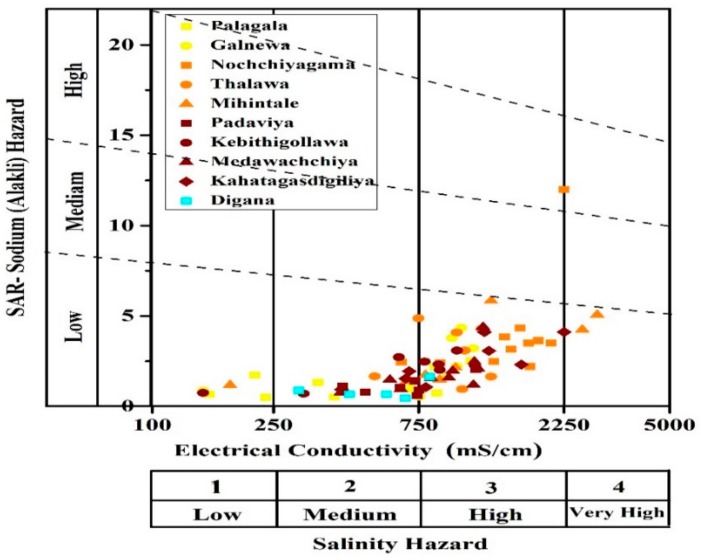
Salinity hazard diagram for collected water samples (dry season).

Sodium adsorption ratio (SAR), along with pH, characterizes the salt-affected soils. It is an easily measured property that gives information on the comparative concentrations of Na^+^, Ca^2+^, and Mg^2+^ in soil solutions. The equation (4) used to calculate SAR is given, as follows:

(4) $$SAR=\;\frac{\left[Na^{+}\right]}{\sqrt{\frac{1}{2}\left(\left[Ca^{2+}\right]+[Mg^{2+}]\right)}}$$

where the [Na^+^], [Ca^2+^], and [Mg^2+^] concentrations are the milliequivalent weight of sodium, calcium, and magnesium ions in the soil solution. To understand the degree of suitability of groundwater for agricultural purposes, the data were plotted on sodium hazard vs. salinity hazard diagram that was proposed by US Salinity Laboratory Staff [33]. Almost all of the samples show only a low alkali hazard (Figure 5). However, most of the samples show a medium to high salinity hazard. This may probably be due to the accumulation of sufficient groundwater during rainy periods and leaving much salt during the dry periods where water is insufficient to leach them out or due to the dissolution of Ca-bearing minerals in the circulating groundwater [34]. Samples that were collected from the control region Digana (closer to wet zone when compared to other zones) are characterized with low sodium absorption ratio (SAR) and, hence, less vulnerable to salinity hazards.

### 3.2. Characterization of DOC Content

Natural organic matter in water is ubiquitous and it acts as a precursor in forming disinfectant by products or carcinogens [24]. Recently, our research group have shown that the organic factions of molecular weights range 900–1800 Da are enriched in the groundwater of high CKDu prevalence zones [11]. Therefore, investigations into this direction are noteworthy. However, in our studies, we only confined the dissolved organic carbons (DOC). The dissolved organic carbon (DOC) concentrations from natural/unpolluted groundwater are typically below 4 mg/L, concentrations above this level generally indicate anthropogenic influences and/or contamination issues that pose serious threats to water safety [35]. Fulvic acid and humic substances contribute to a large portion of the organic matter reservoir in natural waters [36]. These soil humus and/or terrestrial and aquatic plant derived humic substances contribute to one third to over half of the DOC in natural waters [37]. Most of the samples in CKDu prevailing areas reported relatively higher DOC values, and their DOC concentrations are generally higher in the rainy season when compared to the dry season. The majority of the sampled wells are in the vicinity agricultural lands, and there is high probability of mixing terrestrial organic matter with the infiltrated water.

**Figure 6:**
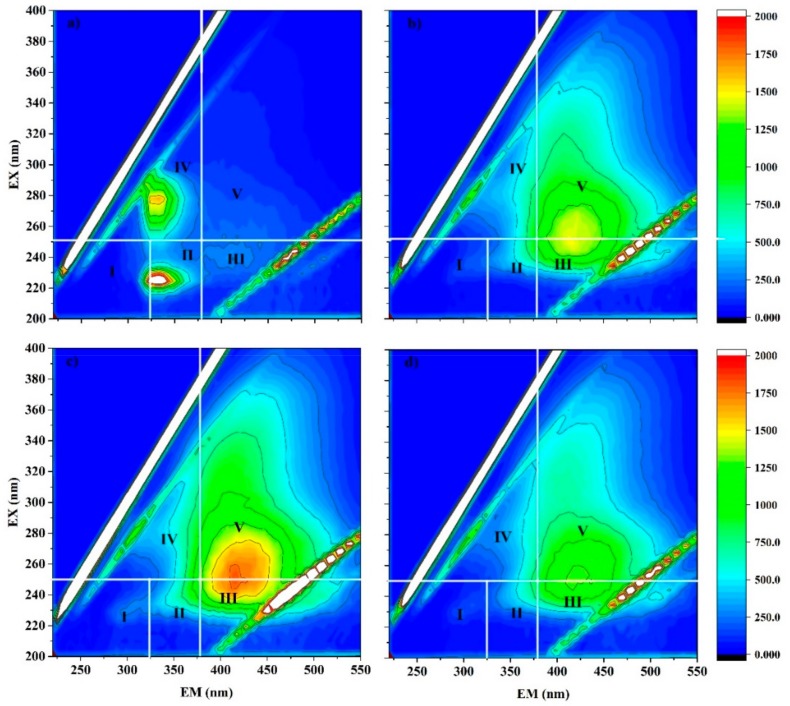
Fluorescence excitation–emission matrixes (EEM) of the selected water samples from the wet season. Region I: Aromatic Protein I; Region II: Aromatic Protein II; Region III: Fulvic acid-like substances; Region IV: Soluble microbial by-product-like substances; Region V: Humic acid-like substances. (**a**) Sample 5, (**b**) sample 9, (**c**) sample 21 and (**d**) sample 34.

The Excitation and Emission Matrices (3DEEM) of water samples given peaks in four main regions (Figure 6): the first in Region II: Aromatic Protein II, Ex 220–230/Em 325–350; the second in Region III: Fulvic acid-like substances, Ex 230–250/Em 400–425; the third in Region IV: Soluble microbial by-product-like substances, Ex 260–280/Em 325–350; and, the forth in Region V: Humic acid-like substances Ex 280–300/Em 400–425. In some samples, a combined peak of fulvic-like substances and humic-like substances appear around Ex 240–260/Em 400–450. The number and the shape of the peaks were different from sample to sample and there were also slight changes of peaks between seasons at the same sample point. However, generally, only the above-mentioned types of peaks appeared in EEM spectra and, as a whole, the samples contained the following organic constituents in decreasing order: fulvic acid-like substances >humic acid-like substances > aromatic protein II > soluble microbial by-product-like substances. Accordingly, fulvic like substances were the most common type of DOC present in the groundwater samples.

The molecular weight (MW) distribution of DOC of groundwater samples gave peaks only in the 100–3000 Da region (Figure S5). There were few distinguishable peaks in the regions of 200 Da, 300 Da, 500–600 Da, 700–900 Da, and 1000–1200. From these data, it was difficult to establish a relationship between the DOC concentration and with the different molecular fractionations that resulted from the EEM spectra, and further study identifying the structural elucidations of DOC molecular fractions is required.

### 3.3. Water Quality Index (WQI)

**Figure 7:**
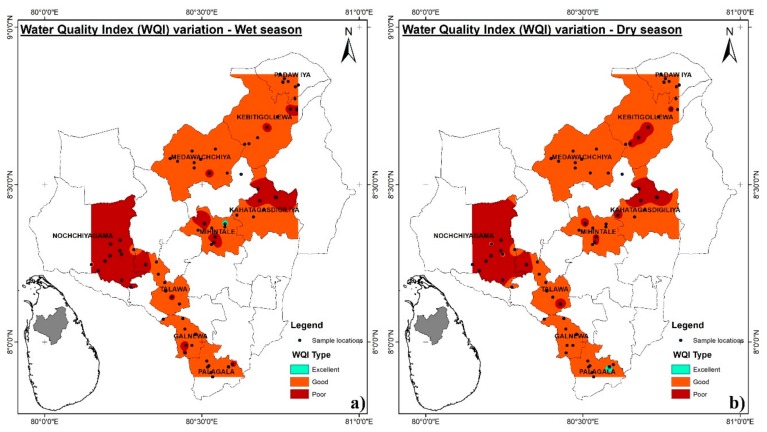
Water Quality Index (WQI) map of groundwater in the study area in the wet season (**a**) and dry season (**b**).

As discussed in Section 3.1., in terms of water treatment perspectives, the geochemical classification that is based on the Piper diagram is of limited value. For example, it has given no provision to incorporate DOC or other species that are relevant to a specific requirement (in our case membrane fouling). Therefore, the availability of an integrated value that is based on required parameters is more useful. As a first step, we introduced the integrated water quality index (WQI). The sub-groups of water types were categorized, as mentioned in Table S2. The WQI value of our samples never exceeded the 200 limit; therefore, only three types (“excellent”, “good”, and “poor”) are further discussed. Table 2 presents a summary of the WQI values and water types in this study. The WQI ranges from 21.2 to 173.6 and 22.7 to 178.3 in the wet and dry seasons, respectively. Throughout the investigated CKDu areas, only three types of water (excellent, good, and poor) were present. In the dry season, the WQI values are generally higher than those of wet season, overall water quality was decreased, the presence of “excellent” type water was decreased, and “good” and “poor” type water increased (Figure 7). However, in certain parts of the study area (Figure 7), “good” type water present in the dry season and “poor” type water present in the wet season; based on the reported data this could be attributed to the leaching of pollutants from shallow aquifer zones (specially organic matter) in to the well [22]. In all samples, natural springs possessed “excellent” or “good” (close to excellent type) type water in both the wet and dry seasons. Only 3.8% in wet season and 2.6% in dry season of the total water samples were found to be in “excellent” type and over 28.2% in wet season and 30.3% in dry season, respectively, considered to be unacceptable for the consumption and appropriate treatment method is essential. 

### 3.4. Membrane Driven Water Treatment

The water in the study area contains high electrical conductivity (EC), which renders it largely unpalatable. Membrane based treatment methods are successful in the desalination of this kind of water. However, the technology requires application after critically characterizing the source water, otherwise the technology may become unusable. Presently, community scale membrane treatment plants that are based on reverse osmosis (RO) are installed throughout the CKDu prevailing areas [16]. This study developed a WQI values for rapid demarcation of the source to suggest an appropriate treatment methods. The WQI value of a water sample that exactly meets the Sri Lankan standards is 90. Therefore, up to WQI < 100 (good) water can be considered as requiring only minimum treatment. Accordingly, it is recommended that the poor water with WQI > 100 requires desalination to some degree. The EC/TDS in water sample can result from mono or/and divalent ions. If the TDS value largely stems from divalent cations (Ca, Mg), then nanofiltration (NF) membrane technology at 50~100 psi is deemed to be suitable. To treat high TDS water due to mono-valent ions requires treatment by reverse osmosis membrane technology. In both cases, membrane fouling poses a serious issue. Membrane blocking by scaling can often be resolved by intermittently auto-flushing with permeates. On the other hand, dissolved natural organic matter (which represent a large fraction of DOC) are aromatic and aliphatic organic moieties that are identified as potent foulants of membranes [38,39]. It is also noted that organic-bound silica and organic-bound calcium strongly contribute to fouling, while Fe derived compounds are considered for irreversible fouling of NF and RO membranes [17]. Recently, it is noted that natural organic matter (NOM) contributes to both reversible and irreversible fouling on NF, even after pretreatment with the combined coagulation and micro filtration process [40]. Thus, source water monitoring is of the utmost importance in identifying possible foulants in designing appropriate pretreatment prior to membrane treatment, as it can seriously impart treatment plant efficiency. 

Finally, the causative agents for the incidence of CKDu cannot be discretely elucidated. It appears that external environmental factors are multi-faceted. We noted that the DOC concentrations in the CKDu affected areas are high. However, the identification of discrete organic compounds that are present in DOC is not studied. It is urgently requires a systematic detailed characterization of the DOC. Sri Lanka is an agricultural country that extensively uses pesticides for cultivation. However, pesticide data in the CKDu areas are scarce to date. Therefore, DOC characterization requires the identification of organic compounds from both anthropogenic and natural sources separately. Besides, it would be an advantage to expand the study area with a greater number of samples and to also include more tube well samples to demarcate the different aquifer types, i.e., regolith vs. fractured rock aquifers and shallow vs. deep aquifers.

## 4. Conclusions

For the first time in Sri Lanka, the geochemical characteristics of groundwater were assessed in different CKDu prevailing areas with the association of WQI with respect to drinking water sources and an appropriate treatment method. Additionally, this is the first study assessing the suitability of groundwater for drinking purposes in CKDu prevalence regions of the country. An elevated concentration of hardness, fluoride, salinity, DOC levels, and alkaline nature of water were the main issues that were encountered in the sampled groundwater of the areas. According to WQI values, the water samples of excellent category (WQI < 50) can be consumed without treatment. However, only 3.8% in the wet season and 2.6% in the dry season of total water samples were found to be of “excellent” type, respectively; the majority of the water sources in the areas do not qualify for drinking without treatment. At present, almost all communities of the affected areas rely on small scale RO treatment plants for drinking water supply. In agreement with the obtained data of raw water quality, there can be serious concerns of these RO plants in terms of extended period operation, maintenance, and management due to alkaline water with higher concentrations of Ca, Mg, Fe, and DOC will cause serious issues in membrane fouling, as the majority of these plants lack any pretreatment steps. As there is an urgent need in establishing proper long-term drinking water treatment technologies for the area, these findings can be used as a benchmark of raw water quality in the design processes of water supply in the CKD affected area.

## Figures and Tables

**Table 1:** Summary of the hydro geochemical data of two seasons.

CKDu Prevalence		pH		EC (μS/cm)		Alkalinity (mg/L)			Hardness (mg/L)		F^−^ (mg/L)		DOC (mg/L)		Cl^−^ (mg/L)			SO_4_^2−^ (mg/L)		Fe (μg/L)	
		Wet	Dry	Wet	Dry	Wet		Dry	Wet	Dry	Wet	Dry	Wet	Dry	Wet	Dry		Wet	Dry	Wet	Dry
High	Min	6.6	5.7	35.2	147.0	11.2		23.9	29.0	37.9	0.5	0.7	1.4	1.8	6.6		2.5	4.3	1.8	2.3	1.0
	Max	8.8	8.2	2080.0	2250.0	387.0		656.0	525.8	604.5	5.3	6.0	11.3	8.1	579.7		525.5	66.3	53.1	462.4	23.9
	Avg	7.8	7.2	729.4	872.2		242.5	282.2	231.0	280.2	2.3	2.8	6.4	5.4	80.5		69.5	23.7	19.9	168.1	9.7
Moderate	Min	7.2	6.7	149.0	180.0		24.4	43.7	32.0	47.9	0.6	0.6	3.2	4.3	11.3		11.2	7.1	7.5	25.5	7.4
	Max	8.7	8.6	2730.0	2890.0		571.0	819.0	637.3	738.8	5.2	4.7	11.0	10.3	532.6		489.1	97.3	98.5	697.3	38.0
	Avg	7.9	7.4	1200.3	1298.1		295.4	349.0	285.1	345.3	2.5	2.2	5.5	6.3	148.7		140.2	33.9	31.0	224.9	13.9
Mild	Min	7.5	6.4	110.1	147.0		13.0	21.6	19.1	25.6	0.2	0.3	2.5	0.1	8.4		4.5	7.1	3.9	73.0	4.6
	Max	8.2	8.4	1356.0	1133.0		419.0	391.0	348.1	350.4	3.7	3.5	10.0	6.8	184.2		121.2	62.6	38.9	220.3	37.2
	Avg	7.8	7.3	664.0	677.6		228.6	172.6	208.6	205.8	2.1	2.4	6.2	3.9	67.4		44.3	27.3	16.7	146.1	12.1
Non-	Min	7.4	6.5	294.0	303.0		106.0	94.8	99.1	108.3	0.7	0.5	2.9	3.0	7.9		6.6	6.5	6.0	66.7	7.6
Prevalence	Max	8.0	7.7	669.0	815.0		247.5	232.0	250.1	308.0	3.3	2.8	5.5	7.1	37.7		85.8	90.9	82.9	221.0	15.7
	Avg	7.8	7.1	490.4	565.0		156.8	135.4	180.9	233.5	2.0	1.5	3.7	4.2	22.7		36.4	27.9	27.0	142.9	10.1
All	Min	6.6	5.7	35.2	147.0		11.2	21.6	19.1	25.6	0.2	0.3	1.4	0.1	6.6		2.5	4.3	1.8	2.3	1.0
	Max	8.8	8.6	2730.0	2890.0		571.0	819.0	637.3	738.8	5.3	6.0	11.3	10.3	579.7		525.5	97.3	98.5	697.3	38.0
	Avg	7.8	7.3	851.8	956.8		251.6	274.0	240.8	284.3	2.3	2.4	5.9	5.3	95.5		84.3	27.9	23.3	181.2	11.6

CKDu: Chronic Kidney Disease of unknown etiology.

**Table 2:** Water quality index (WQI) range and percentage of different water types.

CKDu Prevalence	WQI Range	Water Type	Percentage of Samples in Season	
			Wet Season	Dry Season
All areas	<50	Excellent water	3.8	2.6
	50–100	Good water	68.0	67.1
	100–200	Poor water	28.2	30.3
Mild	<50	Excellent water	11.8	6.3
	50–100	Good water	70.6	93.8
	100–200	Poor water	17.6	0.0
Moderate	<50	Excellent water	3.8	3.7
	50–100	Good water	50.0	48.1
	100–200	Poor water	46.2	48.1
High	<50	Excellent water	0.0	0.0
	50–100	Good water	80.0	69.7
	100–200	Poor water	20.0	30.3

CKDu: Chronic Kidney Disease of unknown etiology.

## Supplementary Materials

The following are available online at https://www.mdpi.com/1660-4601/16/10/1698/s1, Figure S1: (**a**) Districts of Sri Lanka and their CKDu prevalence with over lapped climatic zones based on average annual rainfall and temperature. (**b**) Divisional secretariats of Anuradhapura district and their CKDu prevalence (data source renal registry Ministry of Health Sri Lanka), water samples collected locations are marked. Selected weather stations of (c1) Anuradhapura, (c2) Kandy and (c3) Hambantota temperature (maximum and minimum) variation from 1985 to 2017, Table S1: Assigned and relative weight for WQI computation with SL standards, units of all parameters are in mg/L except pH, Table S2: Classification of groundwater according to Water quality index (WQI), Table S3: Representative hydro geochemical data of analyzed samples, Table S4: Comparison of water quality of different sources (dug wells, tube wells and springs), Figure S2: Gibbs plot of groundwater samples from different CKDu prevalence areas in dry season, Figure S3: (**a**) Fluoride and (**b**) hardness variation over the study area in the wet season, Figure S4: Piper trilinear diagrams showing the different chemical types of groundwater in dry season, (**a**) all samples, (**b**) samples of mild CKDu prevalence areas, (**c**) samples of moderate CKDu prevalence areas and (**d**) samples of high CKDu prevalence areas, Figure S5: Apparent MW distribution of all collected water samples.
