# Supplementary material for: Assessment of Groundwater Quality in CKDu Affected Areas of Sri Lanka: Implications for Drinking Water Treatment
Source: Int J Environ Res Public Health. 2019 May 14;16(10):1698. doi: 10.3390/ijerph16101698 (PMC6572134; doi:10.3390/ijerph16101698)
Supplement: Supplementary file 1 [file ijerph-16-01698-s001.pdf]

Supplementary Documents

Assessment of Ground Water Quality in CKDu Affected Areas of Sri Lanka: Implications for Drinking Water Treatment

Titus Cooray[1, 2, 3, 4], Yuansong Wei[1, 2, 3, *], Hui Zhong [1, 2], Libing Zheng[1, 2], Sujithra K. Weragoda[5], Rohan Weerasooriya[6]

[1] State Key Joint Laboratory of Environmental Stimulation and Pollution Control, Research Center for Eco- Environmental Sciences, Chinese Academy of Sciences, Beijing 100085, China; titus@uwu.ac.lk (T.C); yswei@rcees.ac.cn (Y.W); zhonghui1977@163.com (H.Z); cc lbzheng@rcees.ac.cn (L.Z)

[2]Department of Water Pollution Control Technology, Research Center for Eco-Environmental Sciences, Chinese Academy of Sciences, Beijing 100085, China

[3] University of Chinese Academy of Sciences, Beijing 100049, China

[4] Department of Science and Technology, UvaWellassa University, Badulla 90000, Sri Lanka

[5] National Water Supply and Drainage Board, Katugastota 20800, Sri Lanka; skwera7@gmail.com

[6] National Institute of Fundamental Studies, Hanthana Road, Kandy 20000, Sri Lanka; rohan.we@nifs.ac.lk

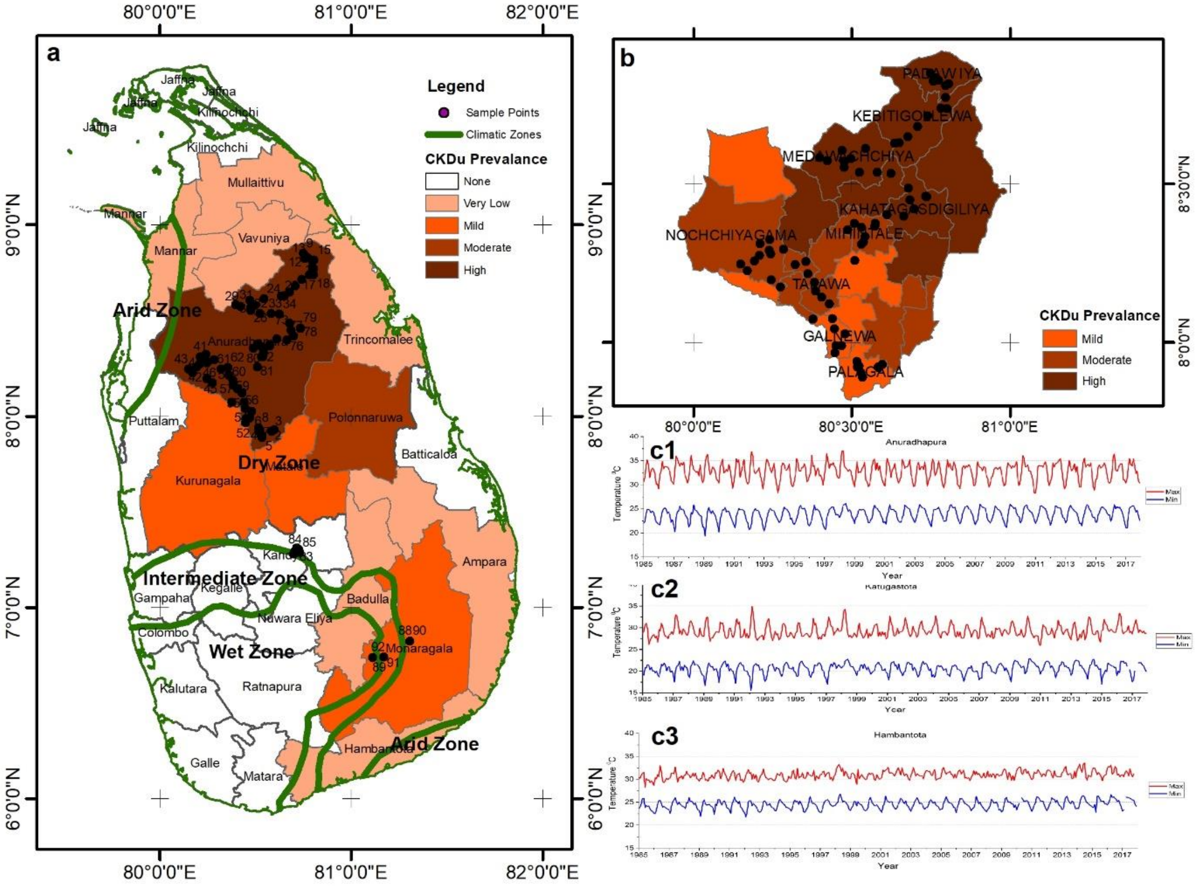

Figure S1. (a) Districts of Sri Lanka and their CKDu prevalence with over lapped climatic zones based on average annual rainfall and temperature. (b) Divisional secretariats of Anuradhapura district and their CKDu prevalence (data source renal registry Ministry of Health Sri Lanka), water samples collected locations are marked. Selected weather stations of (c1) Anuradhapura, (c2) Kandy and (c3) Hambantota temperature (maximum and minimum) variation from 1985 to 2017.

Note – Rainfall data were obtained from Department of Meteorology, Sri Lanka.

Equation S1

- Equation to calculate hardness values based on Ca and Mg concentrations in equivalent of $CaCO_3$ mg/L

  Hardness [mg/L] = 2.497 [Ca, mg/L] + 4.118 [Mg, mg/L]

Table S1. Assigned and relative weight for WQI computation with SL standards,

	Chemical parameter	Sri Lankan Standard	WHO guide line	Weight (w_i)	Relative Weight (W_i)
1	pH	6.5-8.5	6.5-8.5	4	0.0833
2	TDS	500	500	5	0.1042
3	Total Hardness	250	-	2	0.0417
4	Total Alkalinity	200	120	3	0.0625
5	Calcium	100	75	2	0.0417
6	Magnesium	30	50	2	0.0417
7	Sodium	200	200	3	0.0625
8	Fluoride	1	1.5	5	0.1042
9	Chloride	250	250	4	0.0833
10	Sulphate	250	250	4	0.0833
11	Nitrate	50	50	5	0.1042
12	Iron	0.3	0.3	4	0.0833
13	DOC	4*	-	5	0.1042
				Σw_i=48	ΣW_i=1.0000

Units of all parameters are in mg/L except pH.

Table S2. Classification of groundwater according to Water quality index (WQI)

**Class**	**WQI range**	**Water type**
1	<50	Excellent Water
2	50-100	Good Water
3	100-200	Poor Water
4	200-300	Very Poor Water
5	>300	Water Unsuitable for Drinking

Table S3: Representative hydro geochemical data of analyzed samples.

No.	Type	Season	pH	EC (μS/cm)	Alkalinity (mg/L)	DOC (mg/L)	Hardness (mg/L)	F (mg/L)	Cl (mg/L)	SO4 (mg/L)	Na (mg/L)	Ca (mg/L)	Mg (mg/L)	Fe (μg/L)
1	Dug well	Wet	8.0	1143	411	10.0	332.6	2.4	103.5	29.0	143.1	43.1	54.8	147.5
		Dry	7.5	1113	391	0.1	350.4	1.3	74.2	18.3	109.6	44.2	58.5	8.5
4	Dug well	Wet	7.5	259	48	7.6	52.9	3.7	25.4	20.6	35.1	15.9	3.2	82.5
		Dry	6.6	217	43.8	2.5	47.3	3.3	17.4	9.8	27.3	14.6	2.6	6.2
7	Tube well	Wet	7.6	582	169	9.4	243.3	3.2	73.7	14.3	31.8	66.7	18.7	180.5
		Dry	7.2	859	50.1	3.8	328.2	2.5	89.1	7.2	30.6	95.4	21.8	12.7
8	Spring	Wet	7.6	153	25	6.0	43.0	2.9	20.6	10.8	11.8	8.1	5.5	ND
		Dry	7.0	154	21.6	4.9	38.9	2.5	11.6	4.0	9.4	7.2	5.1	14.3
9	Dug Well	Wet	8.1	477	183	11.0	202.2	0.8	14.5	23.9	29.1	51.5	17.9	117.5
		Dry	7.7	500	372	7.6	198.6	3.1	10.9	8.0	25.6	42.5	22.5	2.4
17	Dug well	Wet	7.9	1050	379	10.2	336.3	2.8	94.0	37.8	96.3	47.8	52.9	181.4
		Dry	7.4	1144	622	5.1	386.4	2.5	72.2	31.7	103.5	67.3	53.2	3.1
23	Spring	Wet	7.6	123	11	3.3	29.0	4.9	19.7	5.0	9.5	5.3	3.9	ND
		Dry	5.7	147	23.9	4.9	37.9	2.7	17.6	2.5	10.5	6.6	5.2	2.4
24	Dug well	Wet	7.7	600	194	4.0	214.4	1.0	50.5	21.0	40.4	47.3	23.4	130.7
		Dry	6.8	654	149	4.3	224.9	4.4	33.8	16.1	32.7	50.1	24.3	9.1
35	Dug well	Wet	7.8	544	124	4.9	144.3	3.9	59.8	38.4	61.7	34.5	14.2	86.8
		Dry	7.1	657	221	7.3	178.8	3.2	74.2	32.2	75.1	41.6	18.3	15.4
42	Dug well	Wet	8.7	1981	571	11.0	169.9	5.2	177.2	90.2	430.6	16.7	31.3	25.5
		Dry	8.6	2250	297	8.8	247.7	3.7	296.1	70.0	437.4	16.1	50.6	8.8
43	Dug well	Wet	8.0	1440	233	6.4	332.7	0.6	247.2	16.9	185.9	73.6	36.3	278.5
		Dry	7.4	1614	312	7.8	366.9	0.6	341.9	20.9	191.6	66.0	49.2	10.3
54	Dug well	Wet	7.7	580	276	3.6	169.3	2.4	29.2	14.9	44.0	35.5	19.6	192.8
		Dry	7.3	704	209	4.7	230.5	2.9	16.6	12.8	34.2	61.0	19.0	6.4
62	Dug well	Wet	7.5	776	260	4.0	265.4	1.8	67.8	14.1	42.0	56.7	30.2	245.1

		Dry	7.3	1042	185	6.1	378.7	1.2	88.4	13.7	42.7	90.5	37.2	11.7
73	Dug well	Wet	7.8	2080	266	5.0	525.8	5.3	579.7	15.2	227.4	107.2	62.8	462.4
		Dry	7.6	2250	344	4.2	604.5	6.0	525.5	13.0	233.6	119.4	74.6	18.7
85	Tube well	Wet	7.4	294	106	3.2	99.1	3.3	7.9	6.5	19.7	22.0	10.8	3.9
		Dry	6.5	303	106	3.0	108.3	2.8	6.6	6.0	20.8	23.3	12.2	7.7
	SLS*		6.5-8.5		200		250	1.0	250	250	200	100	30	300
	WHO guideline		6.5-8.5					1.5						

***SLS-Sri Lankan drinking water standard (SLS 614-2013)**

Table S4. Comparison of water quality of different sources (dug wells, tube wells and springs)

CKDu Prevalence	Type of well	pH		EC [µS/cm]		Alkalinity [mg/L]		Hardness [mg/L]		F- [mg/L]		DOC [mg/L]		Cl- [mg/L]		SO_4^{2-} [mg/L]		Fe (µg/L)	
		Wet	Dry	Wet	Dry	Wet	Dry	Wet	Dry	Wet	Dry	Wet	Dry	Wet	Dry	Wet	Dry	Wet	Dry
High	Dug	7.9	7.2	754.0	883.2	244.6	297.2	234.9	282.8	2.3	2.8	6.7	5.6	85.4	71.3	25.6	21.1	165.2	9.5
	Tube	7.8	6.8	712.3	762.0	226.9	212.8	200.4	254.1	1.8	3.0	5.2	4.8	60.5	73.9	13.7	13.9	182.7	12.0
	Spring	7.6	5.7	123.0	147.0	11.2	23.9	29.0	37.9	4.9	2.7	3.3	4.9	19.7	17.6	5.0	2.5	ND	2.4
Moderate	Dug	7.9	7.4	1191.8	1301.6	291.1	349.8	280.4	342.6	2.5	2.2	5.5	6.3	149.1	141.0	34.3	31.3	220.0	13.7
	Tube	7.8	6.9	1306.5	1254.5	348.8	338.5	342.8	379.6	2.4	1.8	5.0	6.6	144.1	131.8	28.2	27.4	286.8	17.0
	Spring																		
Mild	Dug	7.8	7.2	682.3	696.2	235.5	188.9	206.4	205.3	2.1	2.5	5.9	4.1	69.9	43.7	28.7	18.3	122.6	12.3
	Tube	7.7	7.8	526.5	547.5	177.0	58.8	224.5	209.6	2.0	1.4	8.7	2.0	49.0	48.5	17.1	5.5	145.8	10.8
	Spring	7.5	6.7	131.7	150.5	19.1	37.7	31.1	32.3	1.6	3.0	4.2	4.5	18.5	12.1	8.9	4.5	ND	16.3
SLS* standard		6.5 -8.5						250		1.0				250		250		300	
WHO guideline		6.5 -8.5								1.5									

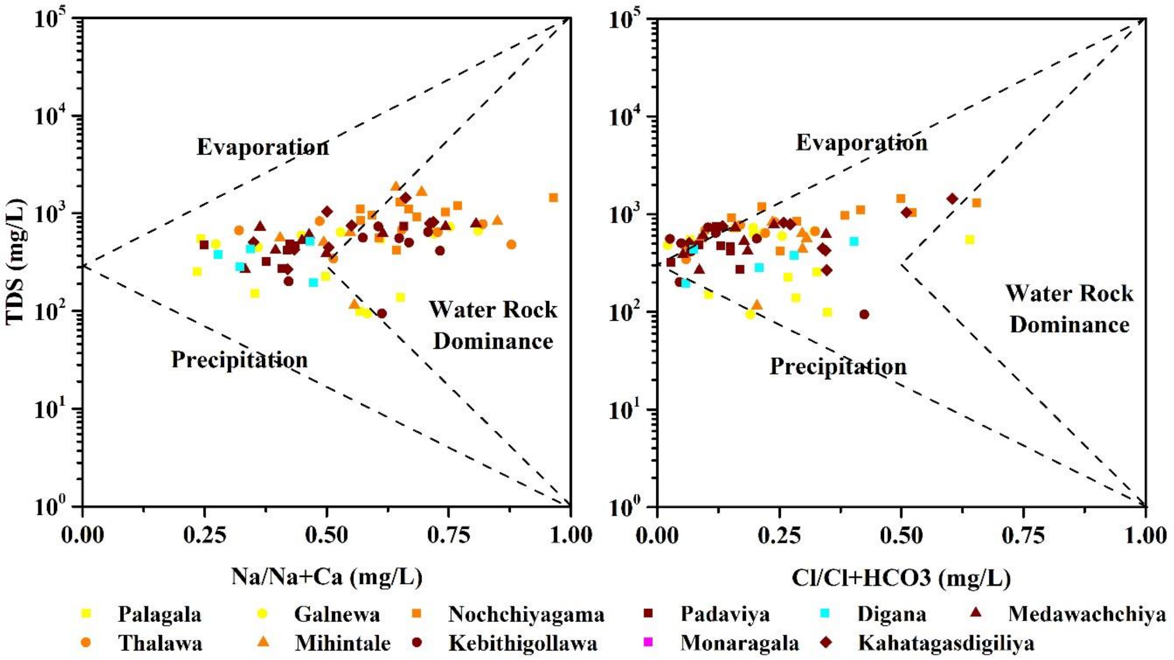

Figure S2. Gibbs plot of ground water samples from different CKDu prevalence areas in dry season.

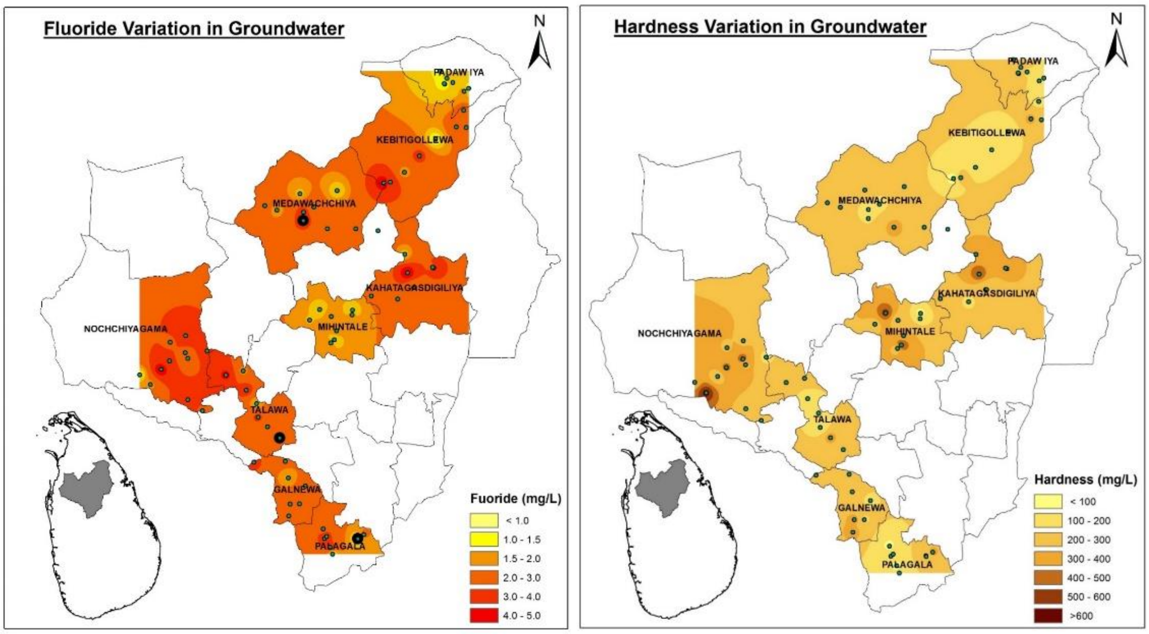

Figure S3. (a) Fluoride and (b) hardness variation over the study area in the wet season

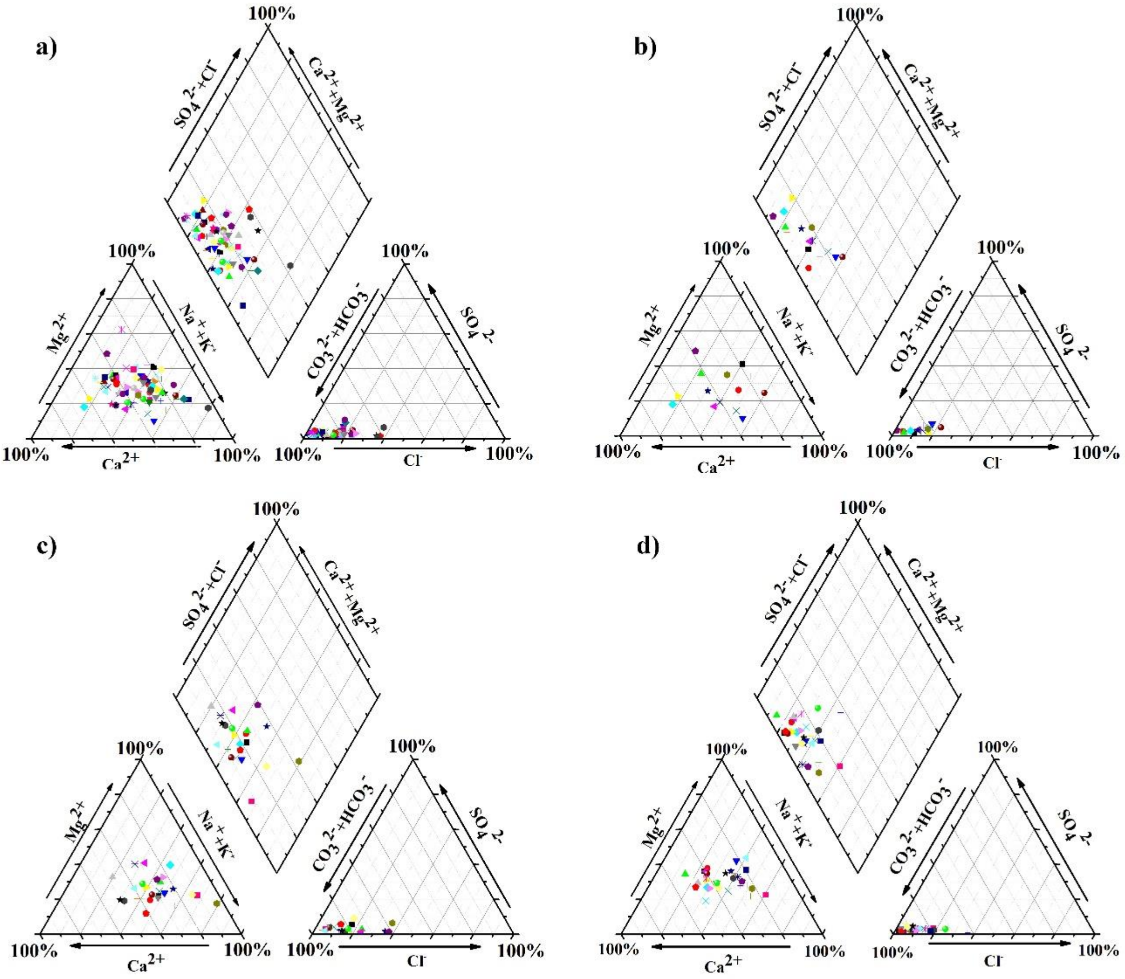

Figure S4. Piper trilinear diagrams showing the different chemical types of ground water in dry season, (a) all samples, (b) samples of mild CKDu prevalence areas, (c) samples of moderate CKDu prevalence areas and (d) samples of high CKDu prevalence areas.

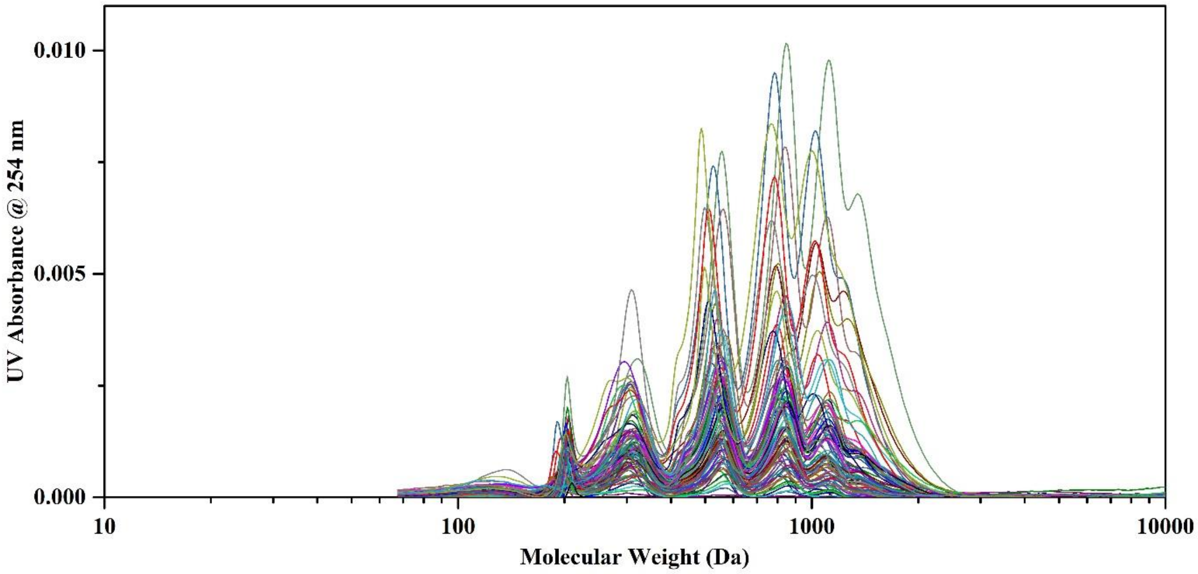

Figure S5. Apparent MW distribution of all collected water samples
